# MERS-Mpro Predictor: A Machine Learning-Based Tool for Rapid Screening of Potential MERS-CoV Main Protease Inhibitors

**DOI:** 10.3390/ijms27094107

**Published:** 2026-05-04

**Authors:** Mebarka Ouassaf, Bader Y. Alhatlani

**Affiliations:** 1 Group of Computational and Medicinal Chemistry, LMCE Laboratory, University of Biskra, Biskra 07000, Algeria; 2 Unit of Scientific Research, Applied College, Qassim University, Buraydah 52571, Saudi Arabia

**Keywords:** MERS-CoV, main protease (Mpro), machine learning, drug discovery, random forest, external validation

## Abstract

The Middle East Respiratory Syndrome coronavirus (MERS-CoV) remains a significant global health concern due to the absence of approved antiviral therapeutics. In this study, we developed a ligand-based machine learning framework to identify potential inhibitors of the MERS-CoV main protease (Mpro) using molecular representations derived from SMILES strings. Multiple classification algorithms, including logistic regression, support vector machines, random forests, and Extreme Gradient Boosting (XGBoost), were systematically evaluated. Model performance was assessed through both internal validation and an external dataset. While several models exhibited strong performance during validation, the Random Forest classifier demonstrated the most robust and consistent generalization, achieving superior predictive performance on the external dataset. To ensure model reliability, a comprehensive validation strategy was implemented, including strict data partitioning to prevent structural overlap, Y-scrambling analysis to eliminate chance correlations, and applicability domain assessment to define the model’s reliable prediction space. The final model was deployed as an interactive web-based application, enabling rapid virtual screening of compounds through single or batch SMILES input, and providing activity predictions along with probability scores and selected physicochemical descriptors. Overall, this study presents a reproducible ligand-based approach for supporting the early-stage identification of potential MERS-CoV Mpro inhibitors.

## 1. Introduction

The Middle East Respiratory Syndrome coronavirus (MERS-CoV) is a zoonotic pathogen belonging to lineage C of the *Betacoronavirus* genus [1]. Since its emergence in Saudi Arabia in 2012, MERS-CoV has caused more than 2600 confirmed human infections and over 900 deaths worldwide, reflecting a case-fatality rate of approximately 35% [2]. Transmission primarily occurs through direct contact with infected dromedary camels, with secondary human-to-human spread, particularly within healthcare settings. Although sustained community transmission has been limited, outbreaks such as the 2015 epidemic in South Korea demonstrated the virus’s potential for rapid international spread, underscoring the urgent need for effective antiviral therapeutics [3,4].

A critical target for therapeutic intervention is the viral main protease (Mpro, also called 3CLpro). This enzyme cleaves the large viral polyproteins pp1a and pp1ab at 11 conserved sites to generate the non-structural proteins essential for genome replication and transcription [5,6]. Mpro is structurally conserved across coronaviruses, featuring a chymotrypsin-like fold (domains I and II) and a distinct helical domain III that is required for dimerization and catalytic activity. Due to its indispensable role in the viral life cycle and the absence of a closely related human homolog, Mpro is considered one of the most attractive drug targets for coronavirus infections [7,8].

Notably, the MERS-CoV Mpro shares high structural and mechanistic similarity with the proteases of SARS-CoV and SARS-CoV-2, both of which have been extensively studied as drug targets [9,10,11]. The clinical success of protease inhibitors such as nirmatrelvir (a component of Paxlovid) against SARS-CoV-2 highlights the therapeutic viability of targeting this enzyme class. However, compared to its relatives, MERS-CoV suffers from a severe scarcity of experimentally validated inhibitor data, which constrains rational drug design and virtual screening efforts [12]. Many reported inhibitors exhibit suboptimal potency, poor selectivity, or unfavorable pharmacokinetic and toxicity profiles, emphasizing the need to discover novel scaffolds with improved drug-like properties [13].

In recent years, artificial intelligence (AI) and machine learning (ML) have revolutionized drug discovery by enabling robust predictive modeling of compound activity, drug–target interactions, and ADMET properties [14,15,16]. ML approaches can integrate heterogeneous datasets, exploit structural similarities across related viruses, and generate accurate predictions even from limited experimental data. Previous studies have demonstrated that ML-based classifiers, including ensemble methods and deep learning architectures, can outperform traditional docking-based virtual screening in the precision-driven identification of antiviral candidates [17,18,19].

To address the critical gap in MERS-CoV therapeutic discovery, we present a dedicated machine learning framework for predicting inhibitors of the MERS-CoV main protease. In contrast to approaches that rely on cross-viral data integration, the present study is based exclusively on experimentally validated MERS-CoV compounds, ensuring that the model learns target-specific structure–activity relationships.

A curated dataset was constructed from the ChEMBL database, followed by rigorous preprocessing, standardization, and duplicate removal to ensure data quality and consistency. Multiple machine learning algorithms were systematically benchmarked to evaluate their predictive performance. Among them, the Random Forest model demonstrated the most robust and consistent performance, particularly in terms of generalization on an independent external validation set.

To ensure practical applicability, the final model was deployed as an interactive, publicly accessible web application that enables rapid screening of candidate molecules. The tool provides predictions of antiviral activity along with associated probability scores and selected physicochemical descriptors. This work presents a ligand-based machine learning framework for the prediction of MERS-CoV Mpro inhibitors and demonstrates its applicability for compound prioritization in data-limited settings.

## 2. Results

### 2.1. Chemical Space Characterization and Dataset Diversity

**Figure 1:**
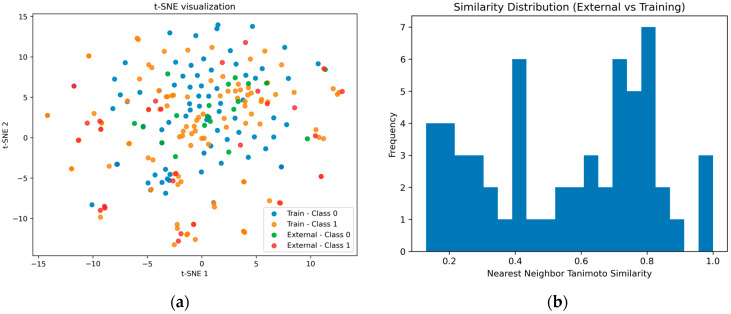
t-SNE embedding of the combined chemical space showing the distribution of training and external compounds (**a**), and nearest-neighbor Tanimoto similarity distribution of external compounds relative to the training set (**b**). The t-SNE plot provides a qualitative visualization of structural overlap, while the similarity histogram quantitatively characterizes the degree of structural relatedness between datasets. The presence of moderate to high similarity values indicates that a substantial portion of the external compounds lies within the chemical space covered by the training data.

We first assessed the chemical diversity and structural relationships within the compiled datasets to evaluate their suitability for robust model training and validation. As shown in Figure 1a, the t-SNE projection indicates that the external validation compounds are largely embedded within the chemical space defined by the training data, supporting their relevance for model evaluation within a similar chemical domain.

A substantial overlap between active and inactive compounds is observed, indicating that structurally similar molecules can exhibit different biological activities. This highlights the inherent complexity of the classification task and suggests that simple linear separation is insufficient, thereby justifying the use of more advanced machine learning approaches to capture these non-linear relationships.

To further quantify the structural relationship between the training and external datasets, a nearest-neighbor Tanimoto similarity analysis was performed. For each external compound, the maximum similarity to the training set was calculated using Morgan fingerprints.

The similarity distribution (Figure 1b) revealed a mean similarity of 0.55 and a median of 0.60, with values ranging from 0.13 to 1.00. Approximately 56.9% of the external compounds exhibited similarity values above 0.5, indicating a moderate to high degree of structural similarity to the training set.

Notably, only a single compound exhibited a similarity value of 1.0, suggesting minimal direct overlap between the training and external datasets at the fingerprint level.

Overall, these findings indicate that the external dataset largely occupies a chemical space overlapping with the training data, while still maintaining a degree of structural diversity. Consequently, the external validation should be interpreted as an evaluation within a related chemical domain rather than a fully out-of-distribution assessment.

Additionally, the presence of peripheral and sparsely distributed points suggests the existence of structurally diverse compounds. This observation further supports the need for applicability domain (AD) analysis to ensure reliable predictions, particularly for compounds located at the boundaries of the chemical space.

### 2.2. Comparative Model Performance

Following the characterization of the chemical space and dataset distribution, we proceeded to train and evaluate multiple machine learning models to assess their predictive performance. As described in the Methods section, molecular descriptors were generated from SMILES representations, and a diverse set of algorithms was selected to capture different learning paradigms, including linear, kernel-based, and ensemble methods.

**Figure 2:**
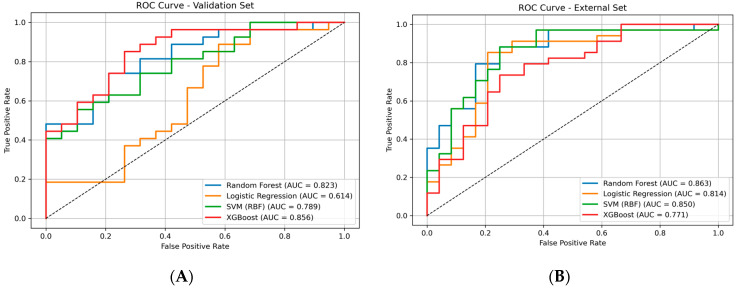
ROC curves comparing model performance on the internal validation dataset (**A**) and the external test dataset (**B**).

The predictive performance of the evaluated models on the validation dataset is summarized in Table 1, with corresponding ROC curves for both validation and external datasets shown in Figure 2A,B.

On the internal validation set, XGBoost achieved the highest overall performance (ROC-AUC = 0.856, PR-AUC = 0.901), indicating that the model is highly effective at distinguishing between active and inactive compounds across different decision thresholds. The high F1-score (0.842) further suggests a good balance between correctly identifying active compounds and avoiding false predictions. Random Forest (ROC-AUC = 0.823) and SVM (ROC-AUC = 0.789) also showed solid performance, meaning that they are reasonably reliable but slightly less effective than XGBoost. In contrast, Logistic Regression showed weaker performance (ROC-AUC = 0.614), indicating limited ability to separate the two classes, likely due to its linear nature.

However, a different trend was observed on the external dataset (Figure 2B). Random Forest achieved the highest ROC-AUC (0.863), suggesting it generalizes well to new, unseen compounds. SVM also performed strongly (0.850), indicating stable predictive behavior. Logistic Regression maintained moderate performance (0.814), meaning that it can still capture some patterns but not as effectively as more complex models. Notably, XGBoost showed reduced performance (ROC-AUC = 0.771), suggesting that while it performs well on known data, it may be less robust when applied to new data.

The ROC curves further support these findings. On the validation set (Figure 2A), XGBoost shows the best separation between classes, while on the external dataset (Figure 2B), Random Forest and SVM maintain more consistent performance across thresholds, indicating better generalization.

In addition to standard metrics, the inclusion of sensitivity, specificity, and Matthews correlation coefficient (MCC) provides a more comprehensive evaluation of model performance, particularly under class imbalance conditions.

Sensitivity values indicate that XGBoost (0.818) is the most effective in correctly identifying active compounds, while Random Forest (0.682) and SVM (0.682) show moderate detection capability. Logistic Regression exhibits slightly higher sensitivity (0.727), suggesting a tendency to identify actives, albeit with lower overall discriminative power.

Specificity values remain relatively consistent across most models (~0.60), indicating a comparable ability to correctly classify inactive compounds. However, XGBoost shows a slightly lower specificity (0.533), reflecting a trade-off between detecting actives and avoiding false positives.

The MCC metric further supports these observations, with XGBoost achieving the highest value (0.369), followed by Logistic Regression (0.327), while Random Forest and SVM show similar moderate performance (0.279). This confirms that XGBoost provides the most balanced classification on the validation set, although its reduced performance on the external dataset suggests potential overfitting.

The difference between validation and external performance highlights the importance of independent testing. While XGBoost appears to learn detailed patterns from the training data, its lower external performance suggests some degree of overfitting. In contrast, Random Forest shows more stable behavior across datasets, making it a more reliable choice for real-world prediction.

To further assess the statistical stability of the selected model, 5-fold cross-validation was performed for the Random Forest classifier. The model achieved a mean ROC-AUC of 0.838 ± 0.036, indicating relatively low variability across folds.

**Figure 3:**
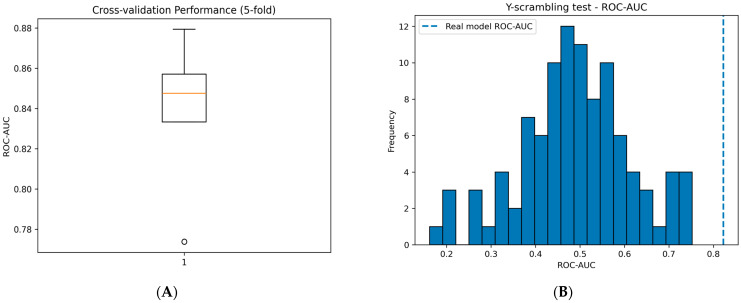
Statistical robustness assessment of the Random Forest model. (**A**) Distribution of ROC-AUC values obtained from 5-fold cross-validation, illustrating the stability of model performance across folds. (**B**) Distribution of ROC-AUC values from the Y-scrambling test, where models are trained on randomly permuted labels. The dashed line represents the performance of the real model, demonstrating that the observed performance is not due to chance.

The distribution of ROC-AUC values across folds is illustrated in Figure 3A using a boxplot representation. This visualization summarizes the central tendency and dispersion of the model performance, where the median, interquartile range, and potential outliers are explicitly shown. The relatively narrow interquartile range and the absence of extreme variability indicate that the model performance is consistent across different data splits.

The predictive performance of the evaluated models on the independent external test dataset is summarized in Table 2. Consistent with the ROC curve analysis, Random Forest achieved the highest ROC-AUC (0.863), confirming its superior generalization capability. SVM also demonstrated strong performance (ROC-AUC = 0.850), indicating stable predictive behavior across unseen data. Logistic Regression showed moderate performance (ROC-AUC = 0.814), while XGBoost exhibited a noticeable decrease (ROC-AUC = 0.771), further supporting the observation of reduced generalization compared to its validation performance.

In terms of threshold-dependent metrics, the models show comparable specificity values, while sensitivity varies, with XGBoost maintaining higher sensitivity but at the cost of reduced specificity. Overall, these results reinforce that Random Forest provides the most balanced and reliable performance on external data.

Overall, this analysis supports the robustness and stability of the Random Forest model, despite the limited size of the dataset.

### 2.3. Model Robustness Assessment Using Y-Scrambling

To further evaluate the robustness of the developed Random Forest model and ensure that its predictive performance is not driven by chance correlations, a Y-scrambling (randomization) test was performed [20]. In this procedure, the activity labels were randomly permuted multiple times (*n* = 100), and the Random Forest model was retrained on each randomized dataset. Detailed results from all Y-scrambling iterations are provided in the Supplementary Materials (Table S1).

As summarized in Table 3, the real model consistently outperformed the scrambled models across all evaluation metrics. The real model achieved a ROC-AUC of 0.823 and PR-AUC of 0.884, whereas the scrambled models produced values close to random expectation (ROC-AUC ≈ 0.49, PR-AUC ≈ 0.64). Similar trends were observed for accuracy and F1-score, which decreased substantially under randomization, indicating that the model’s performance depends on the true relationship between molecular structure and activity.

The distribution of performance values obtained from the scrambled models is shown in Figure 3B The histogram reveals that most scrambled models cluster around random performance, while the real model lies clearly outside this distribution. This separation provides strong visual evidence that the model is not capturing spurious correlations.

Together, these results demonstrate that the model captures meaningful structure–activity relationships and supports its reliability for predictive applications.

### 2.4. Applicability Domain Analysis

The applicability domain was evaluated using a Tanimoto similarity-based approach, where each external compound was compared to the training set using nearest-neighbor similarity.

**Figure 4:**
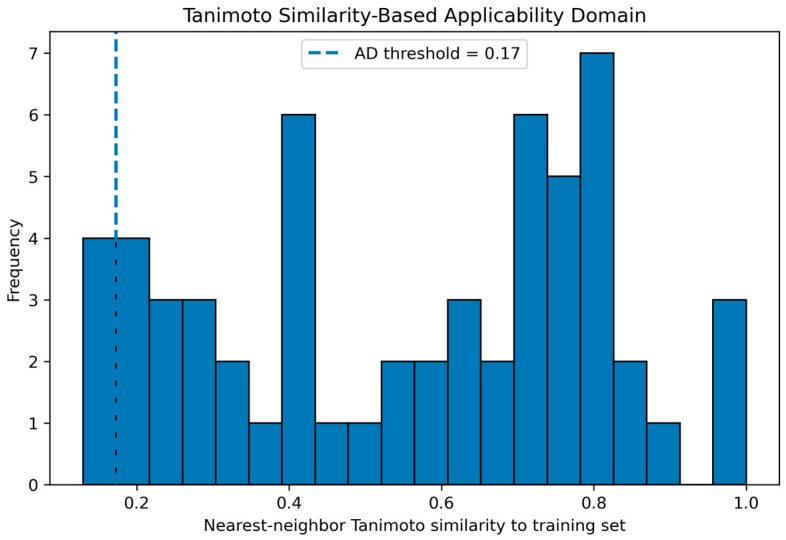
Tanimoto similarity-based applicability domain (AD) analysis. The histogram shows the distribution of nearest-neighbor Tanimoto similarity values between external compounds and the training set. The dashed vertical line indicates the similarity threshold (0.17) used to define the applicability domain. Compounds with similarity values above this threshold are considered within the AD, while those below are considered outside.

As shown in Figure 4, the similarity distribution spans a wide range, with most compounds exhibiting moderate to high similarity to the training set. A threshold of 0.17 was applied to define the applicability domain.

Based on this criterion, the majority of external compounds fall within the applicability domain (93.1%), while a small fraction (6.9%) are considered outside.

Importantly, although a high proportion of compounds lies within the defined domain, the similarity distribution indicates that the external set is not fully independent but rather resides within a related chemical space. Therefore, the reported performance should be interpreted as reflecting model reliability within this domain rather than true out-of-distribution generalization.

### 2.5. Model Interpretability Using SHAP Analysis

To provide mechanistic insight into the model predictions, SHAP (SHapley Additive exPlanations) analysis was performed on the Random Forest model, focusing on the most influential fingerprint features.

**Figure 5:**
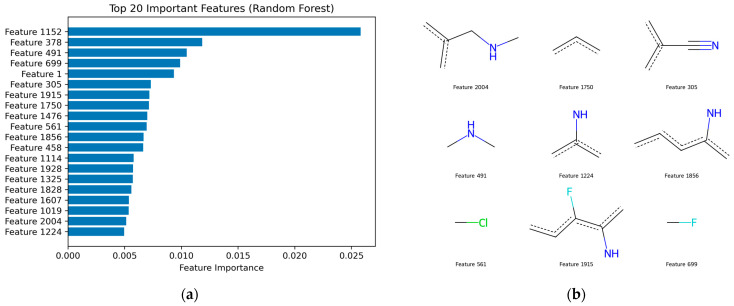
Integrated feature importance and chemical interpretation of the Random Forest model. (**a**) Top 20 most important fingerprint features ranked by their contribution to model predictions. (**b**) Representative molecular fragments corresponding to selected high-importance features, providing structural insight into the learned structure–activity relationships.

As shown in Figure 5a (feature importance), several fingerprint bits exhibit dominant contributions to model predictions, with Features 1152, 378, 491, and 699 among the most influential. However, feature importance alone does not indicate the direction of influence, which was further clarified using SHAP analysis.

**Figure 6:**
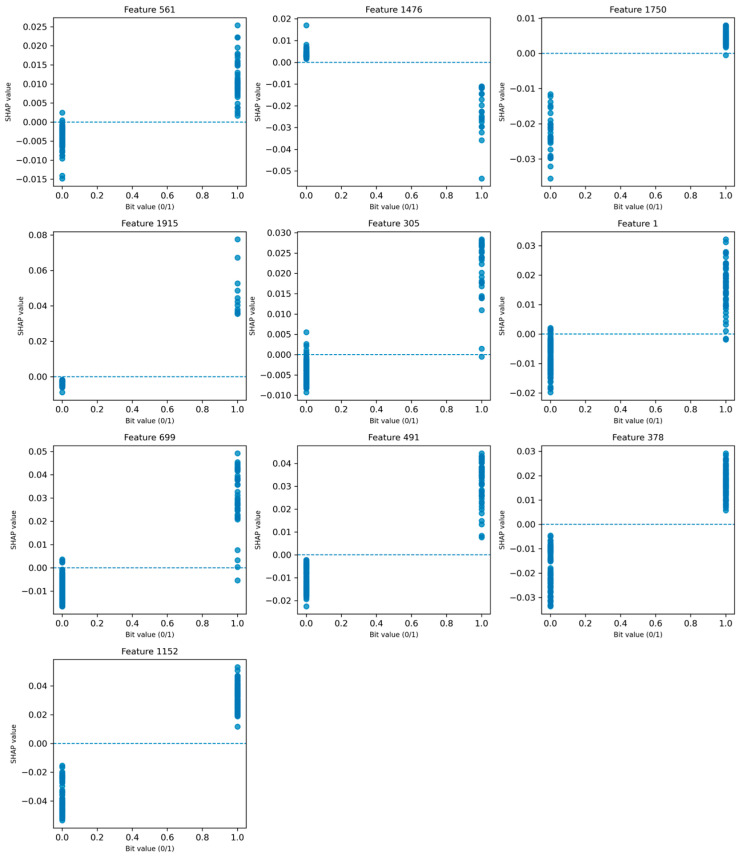
SHAP dependence plots for the top 10 most influential fingerprint features. Each subplot shows the relationship between feature presence (bit value: 0 or 1) and its SHAP contribution to model predictions. Positive SHAP values indicate increased probability of classifying compounds as active, while negative values indicate the opposite.

The SHAP dependence plots (Figure 6) reveal a consistent and interpretable pattern across multiple features. Specifically, for most of the top-ranked features (e.g., Features 561, 699, 491, and 1152), the presence of the feature (bit = 1) is associated with positive SHAP values, indicating an increased probability of classifying compounds as active. Conversely, the absence of these features (bit = 0) generally corresponds to negative SHAP values, reducing the predicted activity.

This clear separation between bit presence and SHAP contribution suggests that the model has learned meaningful structure–activity relationships rather than relying on random correlations. Notably, some features such as Feature 1476 exhibit the opposite trend, where bit presence leads to negative SHAP values, indicating structural motifs associated with inactivity.

To further interpret these fingerprint features chemically, representative molecular fragments corresponding to the most important bits were extracted (Figure 5b). These fragments reveal the presence of chemically meaningful substructures, including nitrogen-containing functional groups (e.g., amines and imine-like motifs), halogen substituents (Cl and F), and unsaturated carbon frameworks.

The consistency between feature importance ranking, SHAP directional effects, and fragment-level chemical interpretation demonstrates that the model captures relevant structural patterns associated with biological activity. This significantly reduces the black-box nature of the fingerprint-based model and enhances its applicability for guiding ligand-based optimization.

### 2.6. Web Tool Deployment for Accessible Screening

**Figure 7:**
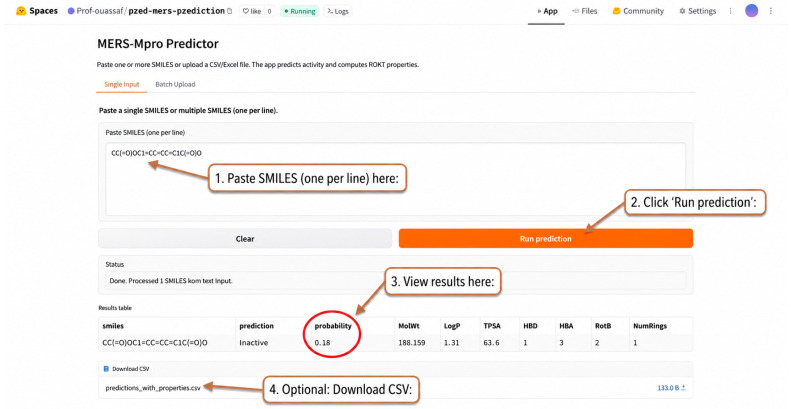
Graphical User Interface of the MERS-Mpro Predictor Web Application for Virtual Screening of MERS-CoV Mpro Inhibitors (https://huggingface.co/spaces/Prof-ouassaf/pred-mers-prediction). (accessed on 10 April 2026).

To facilitate practical application of the developed model, the final Random Forest classifier was deployed as an interactive web application (Figure 7). The tool provides a user-friendly interface that enables rapid screening of compounds without requiring programming expertise.

The application supports multiple input modes, including single SMILES entry, batch input of multiple SMILES strings, and file upload (CSV/Excel) for large-scale screening. For each input molecule, physicochemical descriptors are automatically computed using RDKit, and molecular fingerprints are generated and passed to the trained model for prediction.

The tool returns both the predicted activity class (Active/Inactive) and an associated probability score, reflecting the confidence of the model. Results are presented in an organized table that includes computed molecular properties and can be downloaded as a CSV file for further analysis.

This deployment demonstrates the practical utility of the model and enables its direct use in virtual screening workflows, bridging the gap between model development and real-world application.

### 2.7. Demonstration of Web Tool Usage

To further illustrate the practical utility of the developed model, a small panel of compounds with known or well-recognized pharmacological profiles was submitted to the web application for prediction. As shown in Table 4, the model assigned high probabilities of activity to several established or relevant protease-targeting compounds, including GC-376 (0.95), Nirmatrelvir (0.852), Telaprevir (0.587), and Ritonavir (0.621), all of which were classified as active. In contrast, unrelated or non-protease-targeting compounds such as Paracetamol (0.351), Metformin (0.114), and Warfarin (0.334) were classified as inactive.

Interestingly, some compounds often discussed in antiviral or polyphenolic contexts, such as Disulfiram (0.065), Quercetin (0.185), and Luteolin (0.123), were predicted to be inactive in this setting. These results suggest that the model is not merely favoring broadly known bioactive molecules but rather prioritizes patterns more consistent with the structural features represented in the training data for MERS-CoV Mpro inhibition.

This section provides an illustrative example of how the deployed web tool can be used to generate predictions for user-provided compounds. While not intended as a formal validation experiment, the results demonstrate the model’s ability to prioritize compounds based on their predicted likelihood of activity.

In this context, the tool is designed to support early-stage virtual screening by identifying potentially active candidates for further experimental evaluation, rather than to provide definitive predictions of biological activity

## 3. Discussion

This study presents a machine learning-based framework for the identification of potential inhibitors targeting the Middle East Respiratory Syndrome coronavirus (MERS-CoV) main protease (Mpro), a well-established antiviral target. Multiple classification algorithms, including linear models, support vector machines, and ensemble-based methods, were systematically evaluated to assess their predictive performance across both internal validation and external datasets.

Among the evaluated models, the Random Forest classifier demonstrated the most consistent and robust performance across datasets. While XGBoost achieved the highest performance on the internal validation set, its reduced performance on the external dataset suggests a degree of sensitivity to the training distribution. Random Forest achieved the highest external ROC-AUC and was therefore selected for its superior threshold-independent discrimination, while XGBoost showed stronger performance in threshold-dependent metrics (e.g., accuracy, F1-score, and MCC). This highlights the importance of external validation in model selection, as internal performance alone may not fully reflect real-world predictive behavior [21].

The observed differences between models can be attributed to their underlying learning mechanisms. Ensemble methods such as Random Forest are inherently robust to noise and overfitting, particularly in relatively small and heterogeneous datasets, as they aggregate predictions from multiple decision trees [22,23]. Conversely, models with higher flexibility, such as boosting-based approaches, may capture more complex patterns in the training data but can be more sensitive to distribution shifts, as reflected in their external performance.

Importantly, the substantial overlap between active and inactive compounds observed in the chemical space analysis underscores the intrinsic complexity of the classification task. This indicates that simple linear boundaries are insufficient and supports the use of non-linear machine learning approaches capable of capturing subtle structure–activity relationships [24].

The robustness of the model was further confirmed through Y-scrambling analysis, where randomized models yielded performance close to random expectation, while the real model remained significantly superior. This clear separation indicates that the observed predictive performance is not driven by chance correlations, supporting the validity of the learned relationships.

In addition, applicability domain analysis demonstrated that the vast majority of compounds fall within the model’s reliable prediction space. This suggests that the model operates within a well-defined chemical domain and that its predictions are generally reliable for compounds structurally similar to those in the training dataset.

To further interpret the model predictions at the structural level, fragment-based analysis derived from SHAP values was performed.

The SHAP analysis reveals a clear correspondence with structural motifs commonly found in known viral protease inhibitors [25,26]. In particular, nitrogen-containing functionalities identified in features such as 491, 1224, and 1856 are consistent with amine and amide groups frequently present in clinically relevant protease inhibitors, including Nirmatrelvir and GC-376 [27]. These groups are known to play a key role in hydrogen-bonding interactions within the protease active site.

Similarly, unsaturated carbon frameworks and conjugated systems observed in features such as 1750 and 305 are characteristic of peptide-mimetic backbones and electrophilic functionalities, which are important for binding and, in some cases, covalent interaction with catalytic residues such as Cys145 in coronavirus main proteases [28,29].

Halogen-containing fragments (e.g., feature 561: Cl; feature 699: F) are also notable, as halogen substitution is commonly employed in antiviral drug design to enhance binding affinity, metabolic stability, and lipophilicity. Such substitutions are present in several protease inhibitors, where fluorine atoms in particular contribute to improved pharmacokinetic properties and target engagement [30,31].

Additionally, more complex fragments such as feature 1915, which combine heteroatoms and conjugated systems, resemble structural motifs observed in broad-spectrum protease inhibitors, where multiple interaction types (e.g., hydrogen bonding, hydrophobic contacts, and π-interactions) contribute to effective binding [32].

Overall, these observations suggest that the model captures chemically meaningful patterns that are consistent with known design principles of coronavirus protease inhibitors, supporting its utility for guiding compound prioritization. However, it should be noted that fingerprint-based features do not correspond to uniquely defined chemical fragments, and therefore the interpretation remains approximate.

Furthermore, while the identified fragments are consistent with known protease inhibitor design principles, they do not provide direct mechanistic evidence of binding and should be interpreted as indicative structure–activity patterns rather than definitive structural determinants.

It is important to note that, unlike structure-based approaches such as molecular docking, the present model does not explicitly consider protein–ligand interactions or binding conformations. Therefore, the proposed machine learning approach should be viewed as a complementary tool for rapid compound prioritization rather than a replacement for structure-based methods.

To facilitate practical use, the final Random Forest model was deployed as an interactive web-based application. This tool enables rapid screening of compounds through multiple input modes and provides both activity predictions and associated confidence scores, thereby supporting early-stage prioritization in virtual screening workflows.

While the present model is structure-driven and does not explicitly incorporate experimental assay conditions, it provides a valuable tool for initial compound filtering. Experimental validation remains essential to confirm biological activity under specific conditions.

Finally, although this study focuses on MERS-CoV Mpro inhibition, the overall workflow is generalizable. The combination of SMILES-based representation, machine learning modeling, and rigorous validation strategies can be readily adapted to other therapeutic targets, provided that high-quality curated datasets are available.

### Limitations and Future Direction

While the results of this study are promising, several limitations should be acknowledged.

One important limitation is the heterogeneity of experimental activity data, which includes IC_50_, EC_50_, and percent inhibition values derived from different assay conditions. Although a type-specific thresholding strategy was applied, this may introduce some degree of label uncertainty and affect class boundary definition.

A primary constraint is the limited availability of high-quality, experimentally validated inhibitors targeting MERS-CoV Mpro. This data scarcity restricts the chemical diversity represented in the curated training set and may limit the model’s ability to extrapolate to entirely novel scaffold classes.

Furthermore, although the external validation dataset was strictly separated from the training data at the structural level, it remains moderate in size and occupies a related chemical domain. Evaluation on larger and more structurally diverse compound libraries will be essential to further assess model robustness and generalizability across broader chemical space.

Finally, the current framework is limited to binary activity classification. Future work may extend this approach toward quantitative potency prediction (e.g., IC_50_ regression models) and the integration of additional pharmacokinetic and ADMET-related endpoints. Such extensions would enhance the applicability of the model within practical drug discovery pipelines.

## 4. Materials and Methods

### 4.1. Dataset Collection

The training dataset was compiled from experimentally validated compounds retrieved from the ChEMBL database (European Bioinformatics Institute (EMBL-EBI), Hinxton, UK) [33], including their corresponding identifiers (ChEMBL IDs), molecular structures represented as SMILES strings, and activity measurements such as IC_50_, EC_50_, and percentage inhibition.

All activity values were standardized to a consistent unit (nM) to ensure uniformity across the dataset. Binary activity labels were assigned based on biologically relevant thresholds: compounds with IC_50_ or EC_50_ values ≤ 10 µM were classified as active, while inhibition-based measurements ≥ 50% were also labeled as active. To account for heterogeneity in assay types, a type-specific labeling strategy was applied to ensure consistent interpretation of activity data across different measurement formats.

These thresholds were selected based on commonly adopted criteria in antiviral screening studies, where values ≤ 10 µM are generally considered indicative of biologically relevant activity. Similarly, inhibition values ≥ 50% are widely used as a practical cutoff for defining active compounds in enzymatic assays.

While combining different experimental endpoints may introduce some degree of variability, this approach enables the integration of limited available data for MERS-CoV Mpro and is consistent with common practices in data-scarce QSAR modeling. The potential impact of this heterogeneity on model performance is addressed in the Discussion section.

Duplicate compounds were identified using canonical SMILES representations and removed by retaining a single representative entry per compound, prioritizing the most potent activity measurement.

The full curated dataset was first standardized and cleaned, after which it was split into training and external test sets using stratified sampling to preserve class balance. To ensure structural separation, overlap between the two sets was further verified using canonical SMILES comparison, ensuring that no identical compounds were shared between them.

All curated datasets, including the training and external validation sets with associated ChEMBL IDs and activity values, are provided as structured CSV files in the Supplementary Materials (Tables S1 and S2) to ensure transparency and reproducibility.

The final training dataset consisted of 182 compounds, including 106 active and 76 inactive molecules. The independent external validation set comprised 58 compounds, with 34 active and 24 inactive molecules. 

### 4.2. Data Cleaning and Standardization

All datasets were processed using a standardized preprocessing pipeline. Column names were normalized, and records lacking valid SMILES strings or activity labels were excluded. SMILES strings were validated and canonicalized using RDKit (v2023.09.1) to ensure structural consistency.

The curated dataset was split into internal (training/validation) and external test sets using stratified sampling to preserve class balance. The external test set was strictly excluded from all stages of model development.

### 4.3. Feature Representation

Molecular features were generated using circular fingerprints (Morgan fingerprints) with a radius of 2 and a fixed length of 2048 bits. Fingerprints were computed from canonical SMILES using RDKit and used as input features for all machine learning models. This representation captures local atomic environments and structural patterns relevant to biological activity.

### 4.4. Model Development and Evaluation

Four machine learning algorithms were evaluated for binary classification of compound activity: Logistic Regression (LR), Random Forest (RF), Support Vector Machine with a radial basis function kernel (SVM-RBF), and Extreme Gradient Boosting (XGBoost). These models were selected to represent a range of learning paradigms, including linear, kernel-based, and ensemble methods.

Hyperparameter optimization was performed using grid search combined with stratified cross-validation on the training set. Model selection was based on performance using multiple evaluation metrics, including accuracy, F1-score, ROC-AUC, and PR-AUC.

All models were subsequently evaluated on an independent external test set, which was not used during training or model selection, to assess generalization performance. Based on this evaluation, the Random Forest model was selected as the final model due to its superior and more consistent performance on the external dataset.

To further assess the robustness of the selected model, a 5-fold cross-validation analysis was performed exclusively on the Random Forest model. The average ROC-AUC and its standard deviation across folds were calculated to evaluate model stability.

### 4.5. Model Evaluation

#### 4.5.1. Internal Evaluation

Model performance was first assessed using an internal validation strategy, providing an unbiased estimate of generalization within the chemical space represented in the training data. Predictions were evaluated using a default probability threshold of 0.5.

#### 4.5.2. External Validation

Model robustness was further evaluated using an external dataset that was completely excluded from all stages of model development.

#### 4.5.3. Evaluation Metrics

Performance was assessed using both threshold-independent and threshold-dependent metrics. Discriminative ability was evaluated using ROC-AUC and PR-AUC. Threshold-dependent metrics included accuracy, balanced accuracy, sensitivity (recall), specificity, precision, F1-score, and Matthews correlation coefficient (MCC). Specifically, A 5-fold cross-validation was performed, and performance was reported as mean ± standard deviation. This approach provides an estimate of variability and reduces the dependency on a single data split.

#### 4.5.4. Dimensionality Reduction and Visualization

To explore the structural distribution of the dataset and assess chemical space relationships, dimensionality reduction and similarity-based analyses were performed on Morgan fingerprint representations.

Dimensionality reduction for visualization was performed using t-distributed Stochastic Neighbor Embedding (t-SNE), which projects high-dimensional molecular fingerprints into a two-dimensional space for qualitative analysis of chemical similarity and dataset distribution. To quantitatively assess the relationship between the training and external datasets, a nearest-neighbor Tanimoto similarity analysis was conducted. For each compound in the external dataset, the maximum Tanimoto similarity to the training set was calculated based on Morgan fingerprints (radius = 22,048 bits).

The distribution of these nearest-neighbor similarity values was visualized using a histogram, providing insight into the degree of structural overlap between the training and external datasets. This analysis was further used to support applicability domain assessment.

#### 4.5.5. QSAR Applicability Domain Assessment

The applicability domain (AD) was defined using a similarity-based approach in the original high-dimensional Morgan fingerprint space. For each compound in the external dataset, the maximum Tanimoto similarity to the training set was computed based on nearest-neighbor analysis.

A similarity threshold was determined from the distribution of nearest-neighbor similarities, and compounds with similarity values above this threshold were considered within the applicability domain, while those below were classified as outside.

This approach provides a direct measure of structural similarity between training and external compounds, enabling the identification of predictions made within a chemically relevant domain.

#### 4.5.6. SHAP-Based Model Interpretability Methodology

To improve model interpretability and address the limitations associated with black-box models, SHapley Additive exPlanations (SHAP) analysis was performed on the trained Random Forest model. SHAP is a game theory–based approach that quantifies the contribution of each feature to individual predictions.

A TreeExplainer was employed to compute SHAP values for the molecular fingerprint features. Due to the high dimensionality of the feature space, SHAP values were calculated on a representative subset of the dataset to reduce computational cost.

The resulting SHAP values were used to assess global feature importance and visualize the direction and magnitude of feature contributions using summary plots. This analysis provides insights into how specific fingerprint features influence model predictions and supports the interpretability of the model in a medicinal chemistry context.

#### 4.5.7. Web Tool Deployment

The final Random Forest (RF) model was deployed as an interactive web application on the Hugging Face Spaces platform. The backend was implemented in Python and is responsible for loading the trained model.

The user interface was developed using Gradio and supports three input modes: (i) a single SMILES string, (ii) multiple SMILES strings entered as text (one per line), and (iii) batch processing via CSV or Excel file upload.

For each input molecule, RDKit was used to compute physicochemical descriptors, including molecular weight (MolWt), LogP, topological polar surface area (TPSA), and hydrogen bond donor and acceptor counts (HBD/HBA). The molecular fingerprints were then used as input to the trained Random Forest model to generate predictions.

The application returns both the predicted class (Active/Inactive) and the associated probability score, corresponding to the proportion of decision trees voting for the active class. Results are displayed in a tabular format and can be exported as a CSV file for further analysis.

## 5. Conclusions

This study presents a machine learning-based framework for the identification of potential MERS-CoV Mpro inhibitors using ligand-based representations derived from SMILES and molecular fingerprints. By systematically evaluating multiple algorithms, the Random Forest model was selected as the optimal approach, demonstrating consistent performance across both internal validation and independent external testing.

The use of complementary validation strategies, including external validation, Y-scrambling, and applicability domain analysis, supports the reliability of the model and reduces the likelihood of chance correlations. In addition, the deployment of the model as an interactive web-based application facilitates rapid and accessible screening of candidate compounds, enhancing its practical utility in early-stage drug discovery.

However, several limitations should be acknowledged. The dataset size and the heterogeneity of biological activity data (IC_50_, EC_50_, and inhibition percentages) may introduce uncertainty into the modeling process. Furthermore, the model is limited to binary classification and does not explicitly account for experimental conditions or quantitative activity prediction.

Despite these limitations, the proposed framework provides a reproducible and adaptable ligand-based approach that can support antiviral drug discovery, particularly in data-limited scenarios. Future work will focus on expanding the dataset, improving activity modeling, and integrating additional physicochemical and pharmacokinetic descriptors.

## Figures and Tables

**Table 1:** Performance comparison of machine learning models on the validation dataset.

Model	Accuracy	Balanced Accuracy	F1-Score	ROC-AUC	PR-AUC	MCC	Specificity	Sensitivity
Random Forest	0.717	0.664	0.772	0.823	0.884	0.279	0.6	0.682
Logistic Regression	0.674	0.664	0.769	0.614	0.707	0.327	0.6	0.727
SVM (RBF)	0.674	0.641	0.746	0.789	0.864	0.279	0.6	0.682
XGBoost	0.741	0.676	0.842	0.856	0.901	0.369	0.533	0.818

Abbreviations: ROC-AUC—area under the receiver operating characteristic curve, which measures overall discriminative ability; PR-AUC—area under the precision–recall curve, which is particularly informative for imbalanced datasets; F1-score—harmonic mean of precision and recall; Accuracy—proportion of correctly classified instances; Balanced Accuracy—average of sensitivity and specificity, which accounts for class imbalances; Sensitivity (Recall)—proportion of correctly identified active compounds; Specificity—proportion of correctly identified inactive compounds; Precision—proportion of predicted active compounds that are truly active; MCC (Matthews correlation coefficient)—balanced measure of classification quality taking into account true and false positives and negatives, which is suitable for imbalanced datasets.

**Table 2:** Performance comparison of machine learning models on the external test dataset.

Model	Accuracy	Balanced Accuracy	F1-Score	ROC-AUC	PR-AUC	MCC	Specificity	Sensitivity
Random Forest	0.649	0.676	0.766	0.863	0.785	0.369	0.533	0.818
Logistic Regression	0.676	0.664	0.727	0.814	0.726	0.327	0.6	0.727
SVM (RBF)	0.676	0.642	0.750	0.850	0.809	0.306	0.467	0.818
XGBoost	0.703	0.698	0.792	0.771	0.696	0.426	0.533	0.864

**Table 3:** Performance comparison between the real model and Y-scrambled models.

Metric	Real Model	Scrambled (Mean)
Accuracy	0.717	0.500
F1-score	0.772	0.596
ROC-AUC	0.823	0.489
PR-AUC	0.884	0.644

**Table 4:** Predicted activity and probability scores for selected compounds using the deployed web application.

Compound	Prediction	Probability
GC-376	Active	0.950
Nirmatrelvir	Active	0.852
Telaprevir	Active	0.587
Ritonavir	Active	0.621
Disulfiram	Inactive	0.065
Quercetin	Inactive	0.185
Luteolin	Inactive	0.123
Paracetamol	Inactive	0.351
Metformin	Inactive	0.114
Warfarin	Inactive	0.334

## Data Availability

The datasets generated and/or analyzed during the current study are available within the article and its Supplementary Materials. The source code, trained model, and web-based application developed in this study are publicly available at https://huggingface.co/spaces/Prof-ouassaf/pred-mers-prediction (accessed on 10 April 2026). The repository includes the trained Random Forest model, implementation scripts, and the deployed interface used for compound prediction. All experiments were conducted using Python (v3.10) with RDKit and scikit-learn, as described in the Methods section.

## Supplementary Materials

The following supporting information can be downloaded at: https://www.mdpi.com/article/10.3390/ijms27094107/s1.

## Abbreviations

The following abbreviations are used in this manuscript:

AD	Applicability Domain
ADMET	Absorption, Distribution, Metabolism, Excretion, and Toxicity
AI	Artificial Intelligence
ML	Machine Learning
MERS-CoV	Middle East Respiratory Syndrome Coronavirus
Mpro	Main Protease (3C-like protease, 3CLpro)
PR-AUC	Area Under the Precision–Recall Curve
QSAR	Quantitative Structure–Activity Relationship
RF	Random Forest
ROC-AUC	Area Under the Receiver Operating Characteristic Curve
SMILES	Simplified Molecular Input Line Entry System
SVM	Support Vector Machine
t-SNE	t-distributed Stochastic Neighbor Embedding
XGB	Extreme Gradient Boosting (XGBoost)

## References

[1] Chan J.F.W., Lau S.K.P., To K.K.W., Cheng V.C.C., Woo P.C.Y., Yuen K.-Y. (2015). Middle East Respiratory Syndrome Coronavirus: Another Zoonotic Betacoronavirus Causing SARS-Like Disease. Clin. Microbiol. Rev..

[2] Bleibtreu A., Bertine M., Bertin C., Houhou-Fidouh N., Visseaux B. (2020). Focus on Middle East Respiratory Syndrome Coronavirus (MERS-CoV). Méd. Mal. Infect..

[3] de Wit E., van Doremalen N., Falzarano D., Munster V.J. (2016). SARS and MERS: Recent Insights into Emerging Coronaviruses. Nat. Rev. Microbiol..

[4] Kim K.H., Tandi T.E., Choi J.W., Moon J.M., Kim M.S. (2017). Middle East Respiratory Syndrome Coronavirus (MERS-CoV) Outbreak in South Korea, 2015: Epidemiology, Characteristics and Public Health Implications. J. Hosp. Infect..

[5] Chen R., Gao Y., Liu H., Li H., Chen W., Ma J. (2023). Advances in Research on 3C-like Protease (3CLpro) Inhibitors against SARS-CoV-2 since 2020. RSC Med. Chem..

[6] Zagórska A., Czopek A., Fryc M., Jończyk J. (2024). Inhibitors of SARS-CoV-2 Main Protease (Mpro) as Anti-Coronavirus Agents. Biomolecules.

[7] Hu Q., Xiong Y., Zhu G., Zhang Y., Zhang Y., Huang P., Ge G. (2022). The SARS-CoV-2 Main Protease (Mpro): Structure, Function, and Emerging Therapies for COVID-19. MedComm.

[8] Jin Z., Du X., Xu Y., Deng Y., Liu M., Zhao Y., Zhang B., Li X., Zhang L., Peng C. (2020). Structure of Mpro from SARS-CoV-2 and Discovery of Its Inhibitors. Nature.

[9] Chtita S., Belaidi S., Qais F.A., Ouassaf M., AlMogren M.M., Al-Zahrani A.A., Bakhouch M., Belhassan A., Zaki H., Bouachrine M. (2022). Unsymmetrical Aromatic Disulfides as SARS-CoV-2 Mpro Inhibitors: Molecular Docking, Molecular Dynamics, and ADME Scoring Investigations. J. King Saud Univ.—Sci..

[10] Sivani B.M., Venkatesh P., Murthy T.P.K., Kumar S.B. (2021). In Silico Screening of Antiviral Compounds from *Moringa Oleifera* for Inhibition of SARS-CoV-2 Main Protease. Curr. Res. Green Sustain. Chem..

[11] Fouedjou R.T., Fogang H.P.D., Ouassaf M., Daoui O., Qais F.A., Elkhattabi S., Bakhouch M., Belaidi S., Chtita S., Fouedjou R.T. (2022). Targeting the main protease and the spike protein of SARS-CoV-2 with naturally occurring compounds from some Cameroonian medicinal plants: An in-silico study for drug designing. J. Chil. Chem. Soc..

[12] Reina J., Iglesias C. (2022). Nirmatrelvir plus ritonavir (Paxlovid) a potent SARS-CoV-2 3CLpro protease inhibitor combination. Rev. Esp. Quim. Publ. Soc. Esp. Quim..

[13] Gevorgyan S., Khachatryan H., Shavina A., Gharaghani S., Zakaryan H. (2024). Targeting SARS-CoV-2 Main Protease: A Comprehensive Approach Using Advanced Virtual Screening, Molecular Dynamics, and in Vitro Validation. Virol. J..

[14] Ahmad B., Ouahada K., Hamam H. (2026). Machine Learning for Drug-Target Interaction Prediction: A Comprehensive Review of Models, Challenges, and Computational Strategies. Comput. Struct. Biotechnol. J..

[15] Kant S., Deepika, Roy S. (2025). Artificial Intelligence in Drug Discovery and Development: Transforming Challenges into Opportunities. Discov. Pharm. Sci..

[16] Ouassaf M., Alhatlani B.Y. (2025). DeepTargetClass: A Web-Based Platform for Predicting Protein Target Classes of Small Molecules. J. Comput. Aided Mol. Des..

[17] Nguyen T.H., Thai Q.M., Pham M.Q., Minh P.T.H., Phung H.T.T. (2023). Machine Learning Combines Atomistic Simulations to Predict SARS-CoV-2 Mpro Inhibitors from Natural Compounds. Mol. Divers..

[18] Bi X., Wang Y., Wang J., Liu C. (2025). Machine Learning for Multi-Target Drug Discovery: Challenges and Opportunities in Systems Pharmacology. Pharmaceutics.

[19] Ouassaf M., Mazri R., Khan S.U., Rengasamy K.R.R., Alhatlani B.Y., Ouassaf M., Mazri R., Khan S.U., Rengasamy K.R.R., Alhatlani B.Y. (2025). Machine Learning-Guided Screening and Molecular Docking for Proposing Naturally Derived Drug Candidates Against MERS-CoV 3CL Protease. Int. J. Mol. Sci..

[20] Rücker C., Rücker G., Meringer M. (2007). Y-Randomization and Its Variants in QSPR/QSAR. J. Chem. Inf. Model..

[21] Tropsha A. (2010). Best Practices for QSAR Model Development, Validation, and Exploitation. Mol. Inform..

[22] Barreñada L., Dhiman P., Timmerman D., Boulesteix A.-L., Van Calster B. (2024). Understanding Overfitting in Random Forest for Probability Estimation: A Visualization and Simulation Study. Diagn. Progn. Res..

[23] Imani M., Beikmohammadi A., Arabnia H.R. (2025). Comprehensive Analysis of Random Forest and XGBoost Performance with SMOTE, ADASYN, and GNUS Under Varying Imbalance Levels. Technologies.

[24] Dara S., Dhamercherla S., Jadav S.S., Babu C.M., Ahsan M.J. (2022). Machine Learning in Drug Discovery: A Review. Artif. Intell. Rev..

[25] Owen D.R., Allerton C.M.N., Anderson A.S., Aschenbrenner L., Avery M., Berritt S., Boras B., Cardin R.D., Carlo A., Coffman K.J. (2021). An Oral SARS-CoV-2 Mpro Inhibitor Clinical Candidate for the Treatment of COVID-19. Science.

[26] Zhang L., Lin D., Sun X., Curth U., Drosten C., Sauerhering L., Becker S., Rox K., Hilgenfeld R. (2020). Crystal Structure of SARS-CoV-2 Main Protease Provides a Basis for Design of Improved α-Ketoamide Inhibitors. Science.

[27] Vuong W., Khan M.B., Fischer C., Arutyunova E., Lamer T., Shields J., Saffran H.A., McKay R.T., van Belkum M.J., Joyce M.A. (2020). Feline Coronavirus Drug Inhibits the Main Protease of SARS-CoV-2 and Blocks Virus Replication. Nat. Commun..

[28] Dai W., Zhang B., Jiang X.-M., Su H., Li J., Zhao Y., Xie X., Jin Z., Peng J., Liu F. (2020). Structure-Based Design of Antiviral Drug Candidates Targeting the SARS-CoV-2 Main Protease. Science.

[29] Singh J., Petter R.C., Baillie T.A., Whitty A. (2011). The Resurgence of Covalent Drugs. Nat. Rev. Drug Discov..

[30] Gillis E.P., Eastman K.J., Hill M.D., Donnelly D.J., Meanwell N.A. (2015). Applications of Fluorine in Medicinal Chemistry. J. Med. Chem..

[31] Velásquez-Bedoya P.A., Zapata-Cardona M.I., Monsalve-Escudero L.M., Pereañez J.A., Guerra-Arias D., Pastrana-Restrepo M., Galeano E., Zapata-Builes W. (2025). Antiviral Activity of Halogenated Compounds Derived from L-Tyrosine Against SARS-CoV-2. Molecules.

[32] Pillaiyar T., Manickam M., Namasivayam V., Hayashi Y., Jung S.-H. (2016). An Overview of Severe Acute Respiratory Syndrome–Coronavirus (SARS-CoV) 3CL Protease Inhibitors: Peptidomimetics and Small Molecule Chemotherapy. J. Med. Chem..

[33] Bento A.P., Gaulton A., Hersey A., Bellis L.J., Chambers J., Davies M., Krüger F.A., Light Y., Mak L., McGlinchey S. (2014). The ChEMBL Bioactivity Database: An Update. Nucleic Acids Res..

